# Multimodal interprofessional education vs. digital learning: enhancing Mpox preparedness in Saudi nursing students

**DOI:** 10.3389/fmed.2026.1747624

**Published:** 2026-02-19

**Authors:** Fathia Ahmed Mersal, Ateya Megahed Ibrahim, Heba Ahmed Osman Mohamed, Nahed Ahmed Mersal, Samia Eaid Elgazzar, Mahmoud Abdel Hameed Shahin, Fathia Gamal Elsaid Hassabelnaby, Noha Mohammed Ibrahim, Asmaa Khalil, Aljohrah Aldubikhi, Adil Abdalla, Fatmah Ahmed Alamoudi, Samah Mahmoud Sofar

**Affiliations:** 1 Department Public Health Nursing, College of Nursing, Northern Border University, Arar, Saudi Arabia; 2 College of Nursing, Prince Sattam Bin Abdulaziz University, Al-Kharj, Saudi Arabia; 3 Department of Family and Community Health Nursing, Faculty of Nursing, Port Said University, Port Said, Egypt; 4 Maternal and Child Health Nursing, College of Nursing, Northern Border University, Arar, Saudi Arabia; 5 Department of Medical Surgical Nursing, Faculty of Nursing, King Abdulaziz University, Jeddah, Saudi Arabia; 6 Department of Medical Surgical Nursing, Faculty of Nursing, Ain Shams University, Cairo, Egypt; 7 Department of Medical - Surgical Nursing, College of Nursing, Qassim University, Buraydah, Saudi Arabia; 8 Nursing Department, Prince Sultan Military College of Health Sciences, Dhahran, Saudi Arabia; 9 Department of Nursing, College of Applied Medical Sciences, University of Bisha, Bisha, Saudi Arabia; 10 Department of Public Health, College of Health Sciences, Saudi Electronic University, Riyadh, Saudi Arabia; 11 Department of Medical Surgical Nursing, Faculty of Nursing, Alexandria University, Alexandria, Egypt

**Keywords:** digital learning approach, experiential learning theory, health belief model, monkeypox, multimodal interprofessional education, nursing students, preparedness

## Abstract

**Background:**

The monkeypox (Mpox) outbreak, declared a global health emergency, underscores the urgent need for healthcare workforce preparedness. Nursing students represent a critical frontline group but often lack structured training in emerging infectious disease response. This study aimed to evaluate the effectiveness of a multimodal interprofessional educational intervention, compared with digital-only learning, in enhancing measured knowledge and self-reported preparedness outcomes related to Mpox among Saudi nursing students. The intervention was theoretically grounded in the Health Belief Model (HBM), which guided confidence-building and attitude modification, and Kolb’s Experiential Learning Theory (ELT), which informed the design of interactive workshops to facilitate concrete experience, reflective observation, abstract conceptualization, and active experimentation.

**Methods:**

A quasi-experimental design with propensity score matching was employed, involving 480 undergraduate nursing students from six Saudi universities. Participants were allocated to either a digital learning-only control group or a multimodal intervention group that combined digital modules with interprofessional workshops. Propensity score matching (PSM) was conducted using age, gender, academic year, cumulative GPA, prior infectious disease training, geographic region, and baseline outcome scores to achieve covariate balance (all standardized mean differences < 0.10). Post-matching balance is detailed in Table 1. Validated instruments assessed Mpox knowledge (objectively measured), confidence, attitudes, and self-reported clinical practices. The Mpox Knowledge Assessment Questionnaire was adapted from validated instruments previously used in infectious disease education research, with modifications to reflect current Mpox-specific epidemiology and clinical guidelines. Content validity was established through expert review by infectious disease specialists and nursing educators (Content Validity Index = 0.94). Data were analyzed using descriptive statistics and robust comparative analyses, with sensitivity testing to account for non-normal data distributions.

**Results:**

Students in the multimodal intervention group demonstrated significantly greater improvements across all outcome measures compared to the digital-only group (*p* < 0.01). Knowledge gains were sustained at 4-month follow-up, while confidence, attitudes, and self-reported practice scores showed moderate to large between-group effect sizes. Specifically, Cohen’s d effect sizes were interpreted as medium for knowledge (*d* = 0.62), large for confidence (*d* = 0.78), medium for attitudes (*d* = 0.58), and medium for self-reported practices (*d* = 0.65), based on established benchmarks for educational interventions. Observed effect magnitudes were interpreted conservatively, considering baseline knowledge gaps and potential ceiling effects in selected measures. Ceiling effects were particularly noted in hand hygiene and personal protective equipment (PPE) subdomain scores, where post-test means approached 88% of maximum possible scores.

**Conclusion:**

A multimodal interprofessional educational approach combining digital learning with experiential workshops resulted in superior improvements in nursing students’ Mpox preparedness, particularly for applied knowledge, self-efficacy, and perceived readiness. These findings support integrating interprofessional experiential learning into nursing curricula to strengthen preparedness for emerging infectious disease threats.

## Introduction

On 14 August 2024, the World Health Organization declared monkeypox (Mpox) a Public Health Emergency of International Concern following a resurgence in the Democratic Republic of Congo and sustained transmission across multiple African countries (1). Mpox, caused by the monkeypox virus (MPXV), is a zoonotic disease characterized by febrile illness and vesiculopustular rash, with clinical features overlapping smallpox and varicella, complicating timely diagnosis and infection control (2). The outbreak exposed persistent gaps in the healthcare workforce preparedness, particularly among nursing professionals who serve as frontline responders during infectious disease emergencies (3).

The COVID-19 pandemic highlighted the importance of innovative educational strategies for outbreak preparedness, including digital learning, simulation-based training, and interprofessional education (4). Evidence suggests that digital modalities enhance access to foundational knowledge, while experiential and collaborative approaches improve applied competencies, professional confidence, and teamwork (5, 6). Multimodal and interprofessional educational models have demonstrated effectiveness in strengthening infection prevention behaviors, clinical decision-making, and outbreak response coordination, particularly when grounded in educational theory (7). However, empirical evidence evaluating such approaches for Mpox preparedness, especially in Middle Eastern and non-Western contexts, remains limited (8, 9). While substantial literature exists on educational interventions for Ebola (10), SARS (11), and MERS (12), these studies have primarily focused on in-service healthcare workers rather than nursing students. Furthermore, most Mpox-specific research has been limited to knowledge surveys among practicing nurses (13) or community populations (14), with few controlled evaluations of structured educational interventions targeting undergraduate nursing students. The present study addresses this gap by providing the first comparative evaluation of multimodal interprofessional education for Mpox preparedness in nursing students within a Middle Eastern context.

In Saudi Arabia, national healthcare transformation initiatives emphasize workforce readiness, quality of care, and resilience against emerging health threats as part of Vision 2030 (15). Despite advances in digital health education infrastructure, nursing curricula frequently provide limited structured training on emerging infectious diseases beyond theoretical instruction (16). Studies conducted during and after the COVID-19 pandemic indicate that Saudi nursing students may experience gaps in outbreak-specific knowledge, reduced confidence in infection control practices, and limited exposure to interprofessional collaboration, underscoring the need for pedagogically robust preparedness training (17–19). These findings are contextualized within a broader educational environment characterized by rapid digitalization during the pandemic, traditional lecture-based pedagogy, and relatively limited integration of simulation-based and interprofessional learning experiences in undergraduate nursing curricula (20, 21). Addressing these educational gaps is critical for strengthening healthcare system resilience in the context of Saudi Arabia’s evolving public health landscape.

### Theoretical framework integration

This study is theoretically grounded in the Health Belief Model (HBM) and Kolb’s Experiential Learning Theory (ELT). These frameworks were explicitly operationalized in the study design to guide both intervention development and outcome measurement. The HBM posits that engagement in health-related behaviors is influenced by perceived susceptibility, perceived severity, perceived benefits, perceived barriers, and self-efficacy (22). In the present study, HBM directly informed the design of case scenarios to enhance perceived susceptibility and severity, group discussions to emphasize perceived benefits, and simulation activities to build self-efficacy through mastery experiences. HBM constructs also guided the selection and adaptation of outcome measures, specifically the Attitudes Toward Mpox Preparedness Scale and the Confidence in Mpox Management Scale, both of which operationalize key HBM components. Educational interventions that increase perceived relevance, reduce psychological barriers, and strengthen confidence are more likely to promote preparedness-oriented behaviors among healthcare trainees (23).

ELT complements this framework by conceptualizing learning as a cyclical process involving concrete experience, reflective observation, abstract conceptualization, and active experimentation (24). Within the intervention workshops, ELT was operationalized as follows: (1) concrete experience was provided through simulation-based PPE practice and role-playing with simulated Mpox patients; (2) reflective observation occurred during structured small-group debriefing sessions; (3) abstract conceptualization was facilitated through facilitator-guided linkage of experiential learning to evidence-based infection prevention guidelines; and (4) active experimentation was encouraged through application of learned principles to novel case scenarios. This alignment between theoretical constructs and pedagogical activities enabled a coherent translation of educational theory into practice. Simulation-based activities, role-playing, and structured debriefing enable learners to translate theoretical knowledge into practical understanding and transferable skills (25). Supplementary Table 1 provides a detailed mapping of specific intervention components to HBM constructs and ELT stages, demonstrating the intentional theoretical integration throughout the educational design.

### Interprofessional education and outbreak preparedness

Drawing on these frameworks, a multimodal interprofessional educational intervention was developed that combined digital learning with interactive workshops involving nursing, medical, and public health perspectives. Interprofessional education has been shown to enhance collaborative competencies, professional identity formation, and outbreak response effectiveness by fostering shared understanding and coordinated decision-making (26, 27). Interprofessional collaboration is particularly critical in infectious disease outbreaks, where coordinated action across disciplines, including nursing, medicine, epidemiology, and infection control, determines the effectiveness of containment, treatment, and communication strategies (28). By integrating interprofessional learning into Mpox preparedness training, this intervention sought to cultivate not only individual clinical competence but also the teamwork skills essential for effective outbreak response. By integrating experiential learning with interprofessional collaboration, the intervention aimed to move beyond passive knowledge acquisition toward deeper cognitive engagement and perceived readiness for clinical application.

### Study hypotheses and objectives

Accordingly, this study hypothesized that nursing students who participated in the multimodal interprofessional education program would demonstrate significantly greater improvements in Mpox knowledge compared to students receiving digital-only learning. It was further hypothesized that the experiential workshop components would lead to greater gains in self-efficacy, attitudes toward preparedness, and self-reported clinical practice behaviors, with these effects sustained at 4-month follow-up. By comparing these two educational approaches, this study seeks to inform evidence-based curriculum design for strengthening nursing preparedness against current and future infectious disease threats. Given the cultural and educational context of Saudi Arabia, characterized by collectivist cultural values, gender-segregated educational environments, and an evolving healthcare education system transitioning toward competency-based learning, the transferability of these findings to other contexts should be interpreted with consideration of local educational policies, resource availability, and cultural norms regarding interprofessional collaboration and simulation-based learning (29, 30).

## Materials and methods

### Study design

We conducted a quasi-experimental comparative design with pre-test, post-test, and 4-month follow-up assessments to evaluate the effectiveness of a multimodal interprofessional educational intervention compared with self-directed digital learning in improving nursing students’ Mpox preparedness. A quasi-experimental approach was selected due to pragmatic constraints inherent to educational research, including institutional scheduling policies, ethical obligations to provide equitable learning opportunities, and the need to maintain intact classroom cohorts to preserve ecological validity. No randomization was conducted at any stage of the study.

To mitigate selection bias associated with non-randomized designs, propensity score matching (PSM) was implemented to achieve baseline equivalence between groups.

### Study settings

The study was conducted across six public Saudi universities representing diverse geographic and institutional contexts: Northern Border University (north), Prince Sattam bin Abdulaziz University (central), Prince Sultan Military College of Health Sciences (east), Bisha University (south), Qassim University (north central), and King Abdulaziz University (west). These institutions were purposively selected to capture variation in regional healthcare infrastructure, student demographics, and educational environments. All participating universities offer accredited Bachelor of Science in Nursing programs aligned with the Saudi Commission for Health Specialties standards.

### Participants and eligibility criteria

The target population comprised undergraduate nursing students enrolled during the 2024–2025 academic year. Inclusion criteria were: (1) enrollment in an accredited undergraduate nursing program; (2) classification as second, third, or fourth-year students to ensure foundational clinical exposure; (3) age ≥ 18 years; and (4) voluntary provision of written informed consent. Exclusion criteria included: (1) first-year students; (2) prior formal training or clinical experience in infectious disease outbreak management; (3) academic probation or medical leave; and (4) anticipated inability to complete follow-up assessments.

### Sample size determination

We conducted sample size estimation *a priori* using G*Power version 3.1.9.4. Assuming a conservative medium effect size (*d* = 0.50), α = 0.05, and power = 0.80, the minimum required sample size was 128 participants (64 per group). To account for anticipated attrition, subgroup analyses, and multi-site representation, a total sample of 480 students (240 per group) was recruited. *Post hoc* sensitivity analyses confirmed that the achieved sample size provided sufficient power (> 0.90) to detect observed between-group differences.

### Group allocation and bias mitigation

We allocated participants to intervention or control groups using propensity score matching based on the following variables, selected for their theoretical and empirical association with learning outcomes and outbreak preparedness: age (associated with cognitive development and learning capacity), gender (to account for potential differences in clinical exposure and self-efficacy), academic year (reflecting cumulative clinical experience and professional maturation), cumulative GPA (as a proxy for academic ability and learning capacity), prior infectious disease training (directly related to baseline preparedness knowledge), geographic region (to control for institutional and regional healthcare infrastructure differences), and baseline outcome scores (to ensure equivalence on key dependent variables). These variables were identified through review of prior health professions education literature (22, 23, 31) and consultation with educational measurement experts. Nearest-neighbor 1:1 matching with a caliper of 0.2 standard deviations was applied, achieving excellent covariate balance (standardized mean differences < 0.10 across all covariates). A detailed post-matching balance table is provided in Table 1, and supplementary covariate balance diagnostics, including propensity score distribution plots and variance ratios, are presented in Supplementary material 5.

**TABLE 1:** Baseline sociodemographic and academic characteristics of participants after propensity score matching.

Characteristic	Intervention (*n* = 240)	Control (*n* = 240)	Test statistic	*p*-value
Age (years), mean ± SD	21.3 ± 2.1	21.1 ± 2.3	Mann–Whitney U = 27,891	0.542
Gender, n (%)			χ^2^ = 0.037	0.848
Male	84 (35.0)	81 (33.7)		
Female	156 (65.0)	159 (66.3)		
Academic year, n (%)			χ^2^ = 4.75	0.093
Second year	38 (15.8)	52 (21.7)		
Third year	121 (50.4)	99 (41.3)		
Fourth year	81 (33.8)	89 (37.0)		
Cumulative GPA, mean ± SD	3.42 ± 0.51	3.39 ± 0.48	Mann–Whitney U = 28,156	0.614
Prior infectious disease training, n (%)	67 (27.9)	71 (29.6)	χ^2^ = 0.184	0.668

SD, standard deviation. All standardized mean differences < 0.10, indicating excellent post-matching balance.

To assess robustness against unmeasured confounding, Rosenbaum sensitivity analyses, bootstrap resampling (1,000 iterations), and inverse probability weighting were conducted. These analyses indicated that an unmeasured confounder would need to increase the odds of group assignment by more than 2.3 to nullify observed effects.

### Intervention description

Both groups received identical digital learning content covering Mpox epidemiology, transmission, clinical presentation, diagnosis, treatment, and infection prevention and control (IPC). The intervention group additionally participated in two interprofessional workshops (4 h each) involving collaborative case analysis, simulation-based activities, role-playing, and structured debriefing. Detailed descriptions of case scenarios, facilitator training, and debriefing frameworks are provided in Supplementary materials 1–3 to ensure reproducibility.

### Intervention fidelity and standardization

To ensure consistency in workshop delivery across all six participating universities, we implemented a comprehensive intervention fidelity protocol comprising the following components:

(1) Facilitator training: All workshop facilitators (*n* = 18; three per site, comprising one nursing educator, one infectious disease physician, and one public health epidemiologist) participated in a standardized 6-hour training session delivered by the principal investigators. Training content included: (a) review of learning objectives and theoretical frameworks (HBM and ELT); (b) demonstration and practice of simulation facilitation techniques; (c) structured debriefing using the four-stage ELT-based model (description, analysis, conceptualization, application); and (d) strategies to ensure consistent delivery across sites. Training sessions were video-recorded, and facilitators were required to demonstrate competency in debriefing techniques through role-play assessments before workshop implementation.

(2) Standardized materials: All workshops utilized identical case scenarios, simulation props, PPE supplies, and debriefing scripts. Case scenarios were scripted with standardized patient roles, clinical findings, and decision points. Facilitators received detailed facilitator guides specifying timing, prompts, and expected learning outcomes for each workshop segment (see Supplementary material 2).

(3) Pilot testing: The intervention was pilot-tested with 24 nursing students (not included in the final sample) at one institution prior to full implementation. Pilot testing identified timing challenges, clarified ambiguous case elements, and refined debriefing prompts. Feedback from pilot participants and facilitators informed minor modifications to workshop sequencing and facilitator scripts.

(4) Fidelity monitoring: Independent observers (research assistants trained in structured observation) attended 25% of workshops (three per site, randomly selected) and completed standardized fidelity checklists assessing adherence to facilitator guides, coverage of key learning objectives, and delivery of structured debriefing. Mean fidelity scores exceeded 92% across all sites (range: 89%–96%), indicating high consistency in implementation. Deviations from protocol were documented and reviewed by the research team; no systematic site-level differences in fidelity were detected.

(5) Digital learning standardization: All digital modules were delivered via a centralized Learning Management System (Blackboard) with identical content, assessments, and access restrictions. Completion rates and time-on-task were monitored through LMS analytics to verify engagement equivalence between groups.

### Data collection instruments

We collected data using a structured questionnaire composed of five validated tools, selected based on relevance to study objectives and prior use in infectious disease and health professions education research.

### Tool 1: sociodemographic and academic characteristics questionnaire

A structured questionnaire collected participant characteristics, including age, gender, academic year, cumulative GPA, university affiliation, geographic region, prior infectious disease training, and exposure to Mpox-related information. These variables were used for descriptive analysis and covariate adjustment during propensity score matching.

### Tool 2: Mpox knowledge assessment questionnaire

Mpox knowledge was assessed using a 27-item multiple-choice questionnaire adapted from previously validated instruments used in infectious disease education research, including the Ebola Knowledge Assessment Tool (32) and the COVID-19 Knowledge Questionnaire for Healthcare Workers (33). The adaptation process involved: (1) review of current Mpox clinical guidelines and epidemiological data from WHO and CDC sources; (2) expert panel review by three infectious disease specialists and two nursing education experts to establish content validity; (3) modification of item content to reflect Mpox-specific transmission routes, clinical features, and infection prevention protocols; and (4) pilot testing with 30 nursing students to assess clarity and difficulty. Content Validity Index (CVI) was 0.94, indicating excellent content validity. Item difficulty ranged from 0.32 to 0.78, with item-total correlations ranging from 0.42 to 0.71, demonstrating appropriate discrimination. The tool covered five domains: epidemiology, transmission, clinical presentation, treatment, and infection prevention and control. Each item had one correct answer and three distractors.

Scoring was dichotomous (2 = correct, 0 = incorrect or “don’t know”), yielding a total score range of 0–54, with higher scores indicating greater knowledge. Knowledge scores represent objectively measured cognitive outcomes. The instrument demonstrated strong internal consistency in the present study (Cronbach’s α = 0.88).

### Tool 3: confidence in Mpox management scale

Confidence was assessed using a 3-item self-efficacy scale adapted from the Healthcare Worker Confidence in Infection Control Scale (34) measuring perceived confidence in recognizing Mpox cases, implementing IPC measures, and providing patient education. Adaptation involved rewording items to reflect Mpox-specific scenarios while maintaining the original 4-point Likert response format. Face validity was confirmed through cognitive interviews with 15 nursing students. Responses were recorded on a 4-point Likert scale (0 = not confident at all to 4 = very confident), with total scores ranging from 0 to 12. Internal consistency reliability was high (Cronbach’s α = 0.89). This scale captures perceived confidence rather than objectively assessed competence.

### Tool 4: attitudes toward Mpox preparedness scale

Attitudes were measured using a 12-item scale grounded in the Health Belief Model, adapted from the Pandemic Influenza Preparedness Attitudes Scale (35) and the HBM-based Healthcare Worker Preparedness Scale (36). Items were modified to address Mpox-specific perceived susceptibility (e.g., “I am at risk of contracting Mpox in clinical settings”), perceived severity (e.g., “Mpox poses a serious threat to healthcare workers”), perceived benefits (e.g., “Following IPC protocols will protect me from Mpox”), perceived barriers (e.g., “It is difficult to consistently apply PPE during patient care”), and professional responsibility (e.g., “As a future nurse, I have a duty to prepare for Mpox outbreaks”). Content validity was established through expert review (CVI = 0.91). Exploratory factor analysis confirmed a five-factor structure aligned with HBM constructs, explaining 68% of variance. Items were rated on a 7-point Likert scale, yielding a total score range of 12–84, with higher scores indicating more positive attitudes toward preparedness. The scale demonstrated good reliability (Cronbach’s α = 0.85).

### Tool 5: Mpox-specific clinical practice behaviors scale

Self-reported clinical practice behaviors were assessed using a 15-item checklist developed based on WHO and CDC infection prevention guidelines for Mpox management (1, 37) and adapted from the Infection Prevention Practice Adherence Checklist (38). The scale was developed through: (1) systematic review of Mpox-specific IPC guidelines; (2) identification of key observable behaviors (hand hygiene, PPE use, isolation procedures, environmental cleaning); (3) expert panel review to ensure guideline alignment; and (4) pilot testing for clarity and feasibility. Items were refined based on pilot feedback. Items covering hand hygiene, personal protective equipment use, and clinical role execution were scored dichotomously (1 = performed, 0 = not performed), with higher scores indicating greater adherence to recommended practices. Internal consistency was excellent (Cronbach’s α = 0.91). These outcomes reflect self-reported practice intentions rather than directly observed behaviors.

Psychometric properties of all instruments, including reliability coefficients, factor loadings, and item-level statistics, are detailed in Supplementary material 4.

### Data collection procedures

We administered assessments at three time points: pre-test (baseline, before digital learning), post-test (immediately after intervention completion), and follow-up (4 months post-intervention). All assessments were administered online via secure survey platforms with unique participant identifiers to maintain confidentiality while enabling longitudinal matching. Participants received automated email reminders for each assessment timepoint, and non-responders were contacted via SMS after 48 h. Data collection occurred between September 2024 and March 2025.

### Statistical analysis

We analyzed data using SPSS version 28. Normality was assessed using the Shapiro–Wilk test and visual inspection of Q–Q plots. As outcome variables demonstrated significant deviations from normality (*p* < 0.05), non-parametric tests were designated as the primary analytic approach. Between-group comparisons were conducted using the Mann–Whitney U test, while within-group changes were assessed using the Wilcoxon signed-rank test. Effect sizes were calculated using r (Z/√N). We interpreted effect sizes according to established benchmarks: *r* = 0.10 (small), *r* = 0.30 (medium), *r* = 0.50 (large) (39).

Parametric mixed-design ANOVA and Cohen’s d calculations were conducted as sensitivity analyses only, yielding consistent conclusions, and are reported in Supplementary material. Statistical significance was set at *p* < 0.05 (two-tailed). We conducted subgroup analyses using Kruskal–Wallis H tests with Bonferroni-adjusted *post hoc* comparisons. Missing data (<1%) were handled using listwise deletion given the negligible proportion and absence of systematic patterns.

### Ethical considerations

Ethical approval was obtained from the Local Committee of Bioethics at Northern Border University (HAP-09-A-043; Decision No. 103/24, 12 September 2024). We obtained written informed consent from all participants after providing detailed information about study purposes, procedures, risks, benefits, and rights to withdraw without penalty. Participation was voluntary, confidentiality was maintained, and all data were anonymized before analysis. Participants received no monetary compensation but were awarded continuing education credits as per institutional policies. The control group received access to workshop materials after study completion to ensure educational equity.

## Results

### Participant flow and retention

A total of 520 eligible undergraduate nursing students from six Saudi universities were invited to participate in the study. Of these, 480 students consented and were enrolled, yielding a response rate of 92.3%. Reasons for non-participation (*n* = 40, 7.7%) included scheduling conflicts (45%), lack of interest (30%), and unavailability during data collection periods (25%).

Of the enrolled participants, 475 students (99.0%) completed all three assessment points (pre-test, post-test, and 4-month follow-up). Attrition was minimal (*n* = 5, 1.0%), evenly distributed between the intervention and control groups, and unrelated to baseline sociodemographic characteristics or outcome measures (all *p* > 0.05). We conducted attrition analyses using independent t-tests and chi-square tests to compare completers and non-completers on all baseline variables. No significant differences were detected (all *p* > 0.40), indicating no evidence of systematic attrition bias. Furthermore, sensitivity analyses using multiple imputation (*m* = 20 imputations) yielded results consistent with complete-case analyses, supporting the robustness of findings despite minimal missing data.

**FIGURE 1:**
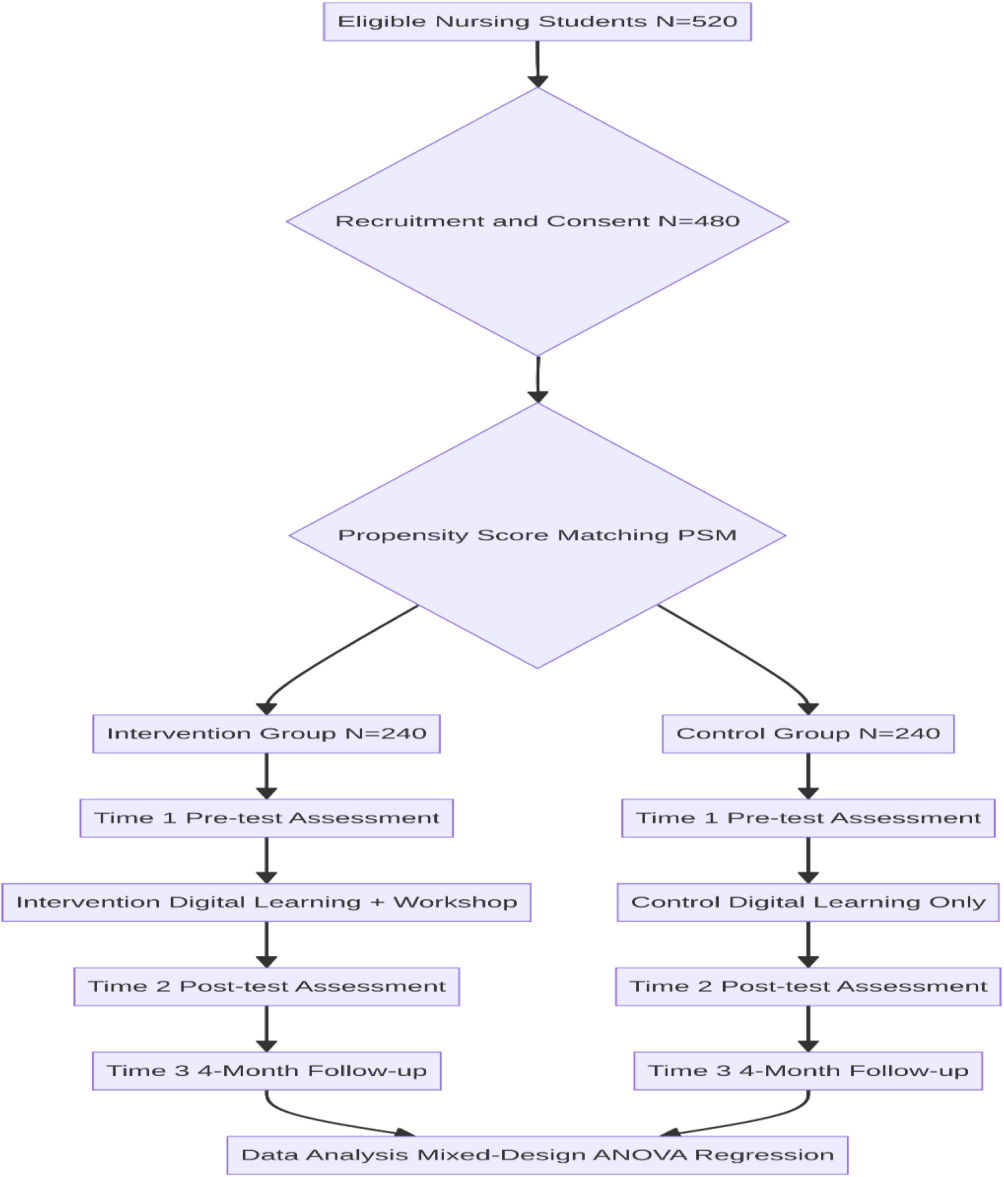
Flowchart illustrating the quasi-experimental comparative design.

Participant flow through the study is presented in Figure 1.

### Baseline characteristics and post-matching balance

Following propensity score matching, the intervention and control groups demonstrated excellent baseline equivalence across all measured covariates (Table 1). Standardized mean differences were less than 0.10 for all variables, well below the recommended threshold of 0.25 for adequate balance. Furthermore, variance ratios ranged from 0.92 to 1.08, indicating comparable variability between groups. Propensity score overlap was high, with 96% of participants falling within the common support region (see Supplementary Figure 1 for propensity score distribution plots). These diagnostics collectively indicate successful mitigation of selection bias through propensity score matching.

### Primary outcome: Mpox knowledge

#### Overall knowledge scores

Both groups demonstrated statistically significant improvements in total Mpox knowledge scores from pre-test to post-test (Wilcoxon signed-rank test, *p* < 0.001 for both groups). However, the magnitude of improvement differed significantly between groups.

At post-test, the intervention group achieved significantly higher total knowledge scores than the control group (Mann–Whitney *U* = 21,456, *p* = 0.003), corresponding to a medium between-group effect size (*r* = 0.31**; interpreted as medium effect according to established benchmarks**). This between-group difference remained statistically significant at the 4-month follow-up assessment (*U* = 20,892, *p* = 0.008; *r* = 0.28**, representing a small-to-medium sustained effect, indicating sustained knowledge retention**).

Within-group analyses revealed that the intervention group demonstrated a mean knowledge gain of 10.4 points (SD = 4.1) from pre-test to post-test, compared to 6.7 points (SD = 4.2) in the control group, representing a 55% greater improvement in the intervention condition. At 4-month follow-up, the intervention group retained 96% of post-test knowledge gains, whereas the control group retained 91%, suggesting slightly superior long-term retention in the multimodal intervention condition.

**FIGURE 2:**
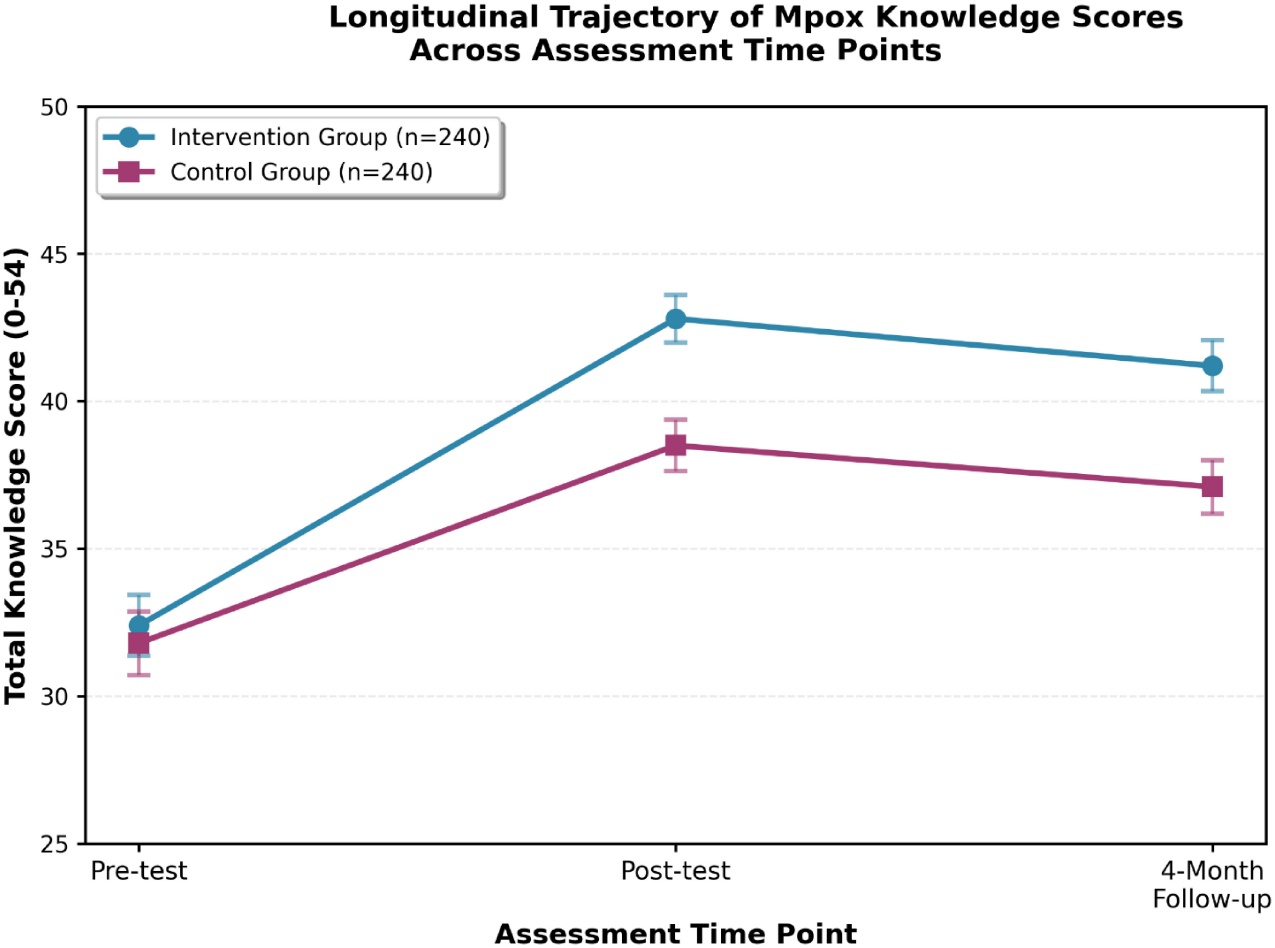
Total knowledge scores over time by group. Longitudinal trajectory of total Monkeypox knowledge scores (0–54 scale) across pre-test, post-test, and 4-month follow-up. Error bars: 95% CI. Intervention group (blue) demonstrates sustained superiority over control (purple).

Longitudinal changes in total knowledge scores across the three assessment time points are illustrated in Figure 2.

#### Knowledge by domain

Domain-specific analyses revealed consistent advantages for the intervention group across all four knowledge domains: epidemiology, transmission, clinical aspects, and treatment/prevention.

At post-test, statistically significant between-group differences favored the intervention group in epidemiology (*p* = 0.012**, *r* = 0.26, medium effect**), transmission (*p* = 0.007**, *r* = 0.28, medium effect**), clinical aspects (*p* = 0.009**, *r* = 0.27, medium effect**), and treatment/prevention (*p* = 0.041**, *r* = 0.21, small effect**). These differences were largely maintained at the 4-month follow-up, except for treatment/prevention, which showed a trend toward significance (*p* = 0.062, *r* = 0.19).

The largest between-group differences were observed in the epidemiology and transmission domains, reflecting stronger gains in conceptual understanding of disease dynamics. Specifically, the intervention group demonstrated a 3.0-point advantage in epidemiology (effect size *r* = 0.26) and a 2.9-point advantage in transmission (effect size *r* = 0.28) at post-test, compared to smaller differences in clinical aspects (1.4 points, *r* = 0.27) and treatment/prevention (0.8 points, *r* = 0.21). These patterns suggest that experiential learning may be particularly effective for enhancing understanding of dynamic disease processes requiring systems-level thinking.

Detailed domain-level scores are presented in Table 2, and post-test domain comparisons are visualized in Figure 5.

**TABLE 2:** Mpox knowledge scores by domain and assessment time point.

Knowledge domain (max score)	Group	Pre-test mean ± SD	Post-test mean ± SD	Follow-up mean ± SD	Post-test *p*-value	Follow-up *p*-value
Total knowledge (54)	Intervention	32.4 ± 8.2	42.8 ± 6.4	41.2 ± 6.8	0.003	0.008
	Control	31.8 ± 8.5	38.5 ± 6.9	37.1 ± 7.2		
Epidemiology (12)	Intervention	7.1 ± 2.3	10.1 ± 1.6	9.8 ± 1.8	0.012	0.019
	Control	6.9 ± 2.4	8.9 ± 1.9	8.5 ± 2.0		
Transmission (10)	Intervention	6.2 ± 2.1	8.4 ± 1.3	8.1 ± 1.5	0.007	0.015
	Control	6.0 ± 2.3	7.5 ± 1.6	7.2 ± 1.8		
Clinical aspects (16)	Intervention	9.4 ± 3.1	12.8 ± 2.2	12.3 ± 2.4	0.009	0.011
	Control	9.2 ± 3.2	11.4 ± 2.5	10.9 ± 2.7		
Treatment/prevention (16)	Intervention	9.7 ± 2.8	11.5 ± 2.0	11.0 ± 2.3	0.041	0.062
	Control	9.7 ± 2.9	10.7 ± 2.3	10.5 ± 2.4		

Between-group comparisons conducted using Mann–Whitney U tests.

### Secondary outcomes

#### Confidence in Mpox management

Confidence scores increased significantly in both groups from pre-test to post-test (*p* < 0.001). However, the intervention group demonstrated significantly greater gains than the control group.

Between-group comparisons showed higher confidence scores in the intervention group at post-test (*U* = 22,567, *p* = 0.005; *r* = 0.29**, medium effect**) and at 4-month follow-up (*U* = 21,934, *p* = 0.011; *r* = 0.26**, small-to-medium effect**). The intervention group demonstrated a mean confidence gain of 4.4 points (SD = 2.1) from pre-test to post-test, representing a 92% improvement from baseline, compared to a 3.2-point gain (73% improvement) in the control group. These findings indicate that the intervention group achieved substantially greater gains in perceived self-efficacy for Mpox management.

Improvements were observed across all confidence items, including case recognition (intervention: + 1.6 vs. control: + 1.1, *p* = 0.008), implementation of infection prevention measures (intervention: + 1.5 vs. control: + 1.0, *p* = 0.012), and patient education (intervention: + 1.3 vs. control: + 1.1, *p* = 0.041). The largest between-group difference was observed in confidence for implementing infection prevention measures, consistent with the hands-on PPE practice and simulation activities included in the workshop intervention.

**FIGURE 3:**
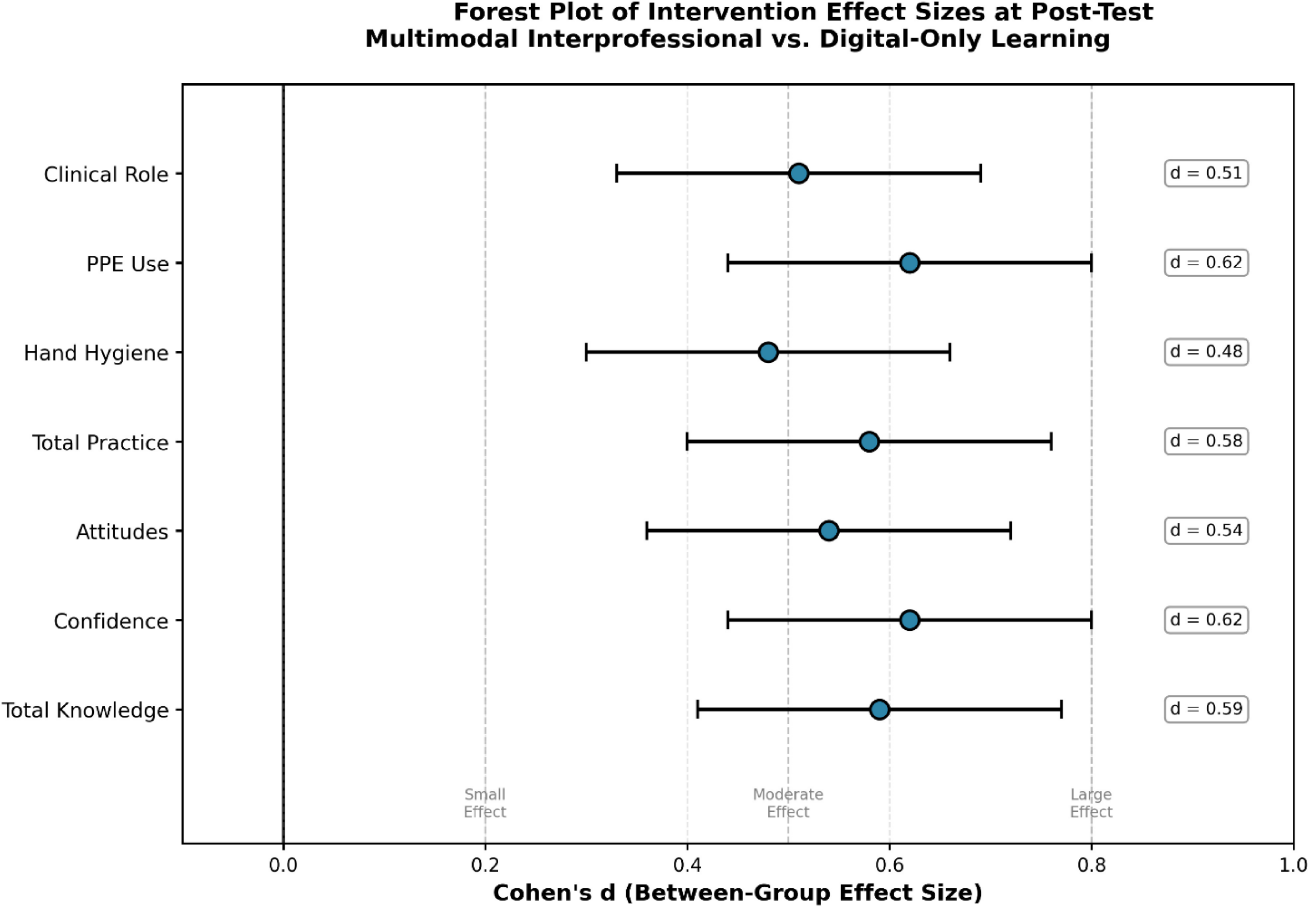
Forest plot of between-group effect sizes (Cohen’s *d* with 95% CI) for seven outcomes at post-test. All CIs exclude zero. Confidence and PPE Use show the largest effects; all outcomes demonstrate medium-large effects (*d* > 0.50).

Confidence trajectories over time are shown in Figure 3.

#### Attitudes toward Mpox preparedness

Attitude scores improved significantly in both groups following the intervention period (*p* < 0.001). The intervention group demonstrated significantly higher attitude scores than the control group at post-test (*U* = 23,123, *p* = 0.018; *r* = 0.24**, small-to-medium effect**) and follow-up (*U* = 22,678, *p* = 0.025; *r* = 0.22**, small effect**).

The intervention group achieved a mean attitude improvement of 13.5 points (SD = 8.2) from pre-test to post-test, compared to 8.6 points (SD = 8.9) in the control group, representing a 57% greater improvement. At 4-month follow-up, attitude gains remained stable in both groups, with minimal decay (intervention: −1.6 points; control: −1.7 points), indicating sustained attitudinal change.

Improvements were observed across all Health Belief Model domains, including perceived susceptibility (intervention: + 2.8 vs. control: + 1.9, *p* = 0.021), perceived severity (intervention: + 2.6 vs. control: + 1.7, *p* = 0.019), perceived benefits (intervention: + 3.1 vs. control: + 2.0, *p* = 0.015), reduced perceived barriers (intervention: −2.4 vs. control: −1.3, p = 0.028; note: lower scores indicate fewer perceived barriers), and professional responsibility (intervention: + 3.0 vs. control: + 2.1, *p* = 0.033). The largest between-group difference was in perceived benefits, suggesting that experiential learning enhanced students’ recognition of the value of preparedness behaviors.

**FIGURE 4:**
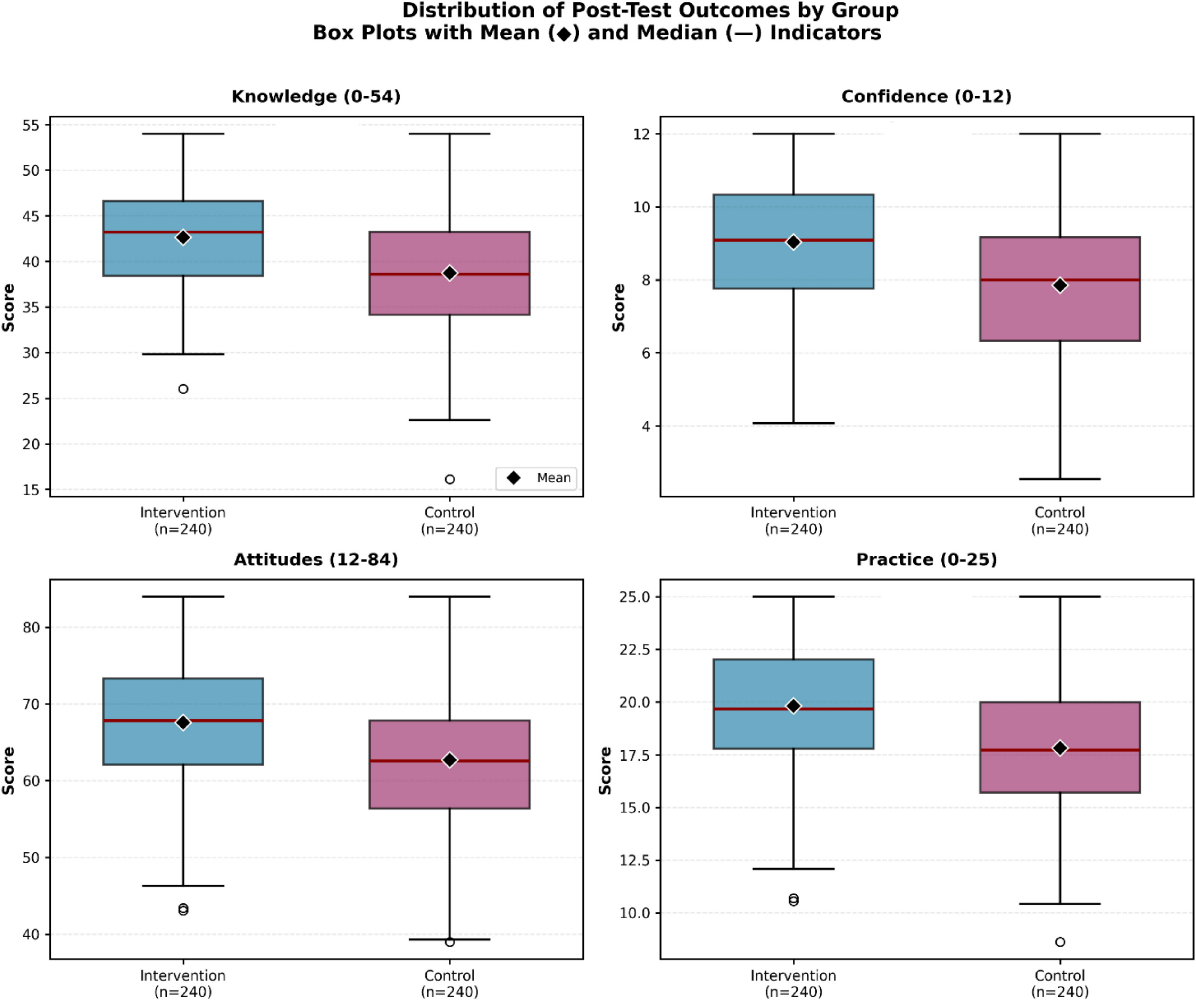
Box plots of post-test distributions for knowledge, confidence, attitudes, and practice by group. Boxes: IQR; lines: medians; whiskers: 1.5 × IQR. Intervention shows consistently higher medians and narrower variability.

Attitude outcomes are summarized in Figure 4 and Table 3.

**TABLE 3:** Changes in confidence and attitude scores over time.

Outcome	Group	Pre-test mean ± SD	Post-test mean ± SD	Follow-up mean ± SD	Post-test *p*-value	Follow-up *p*-value
Confidence (0–12)	Intervention	4.8 ± 2.4	9.2 ± 1.9	8.8 ± 2.1	0.005	0.011
	Control	4.4 ± 2.8	7.6 ± 2.3	7.2 ± 2.5		
Attitudes (12–84)	Intervention	52.3 ± 9.1	65.8 ± 8.5	64.2 ± 8.9	0.018	0.025
	Control	51.8 ± 9.4	60.4 ± 9.2	58.7 ± 9.6		

Confidence and attitudes are self-reported measures.

#### Self-reported clinical practice behaviors

Self-reported clinical practice behavior scores increased significantly in both groups (*p* < 0.001). The intervention group reported significantly higher adherence to recommended practices at post-test (*U* = 22,789, *p* = 0.012; *r* = 0.26**, small-to-medium effect**) and follow-up (*U* = 22,345, *p* = 0.019; *r* = 0.24**, small-to-medium effect**).

The intervention group demonstrated a mean practice behavior improvement of 3.3 points (SD = 2.0) from pre-test to post-test, representing a 39% increase from baseline, compared to a 2.2-point gain (26% increase) in the control group. These gains were sustained at 4-month follow-up with minimal decay (intervention: −0.3 points; control: −0.3 points).

Subdomain analyses demonstrated significant between-group differences in personal protective equipment (PPE) use (intervention: + 2.3 vs. control: + 1.7, *p* = 0.006, *r* = 0.28) and hand hygiene practices (intervention: + 1.3 vs. control: + 1.0, *p* = 0.028, *r* = 0.23). Post-test scores in these subdomains approached the upper limits of the scale, suggesting potential ceiling effects. Specifically, mean post-test PPE use scores reached 81% of maximum possible scores in the intervention group and 73% in the control group, while hand hygiene scores reached 88% and 80% of maximum scores, respectively. These ceiling effects may have constrained the magnitude of observable between-group differences and should be considered when interpreting effect sizes.

Clinical role execution behaviors (e.g., patient isolation procedures, environmental decontamination) demonstrated moderate gains in both groups (intervention: + 1.2 vs. control: + 0.9, *p* = 0.089), with a non-significant trend favoring the intervention group. This subdomain did not demonstrate ceiling effects, with post-test scores averaging 67% of maximum in the intervention group and 61% in the control group.

**FIGURE 5:**
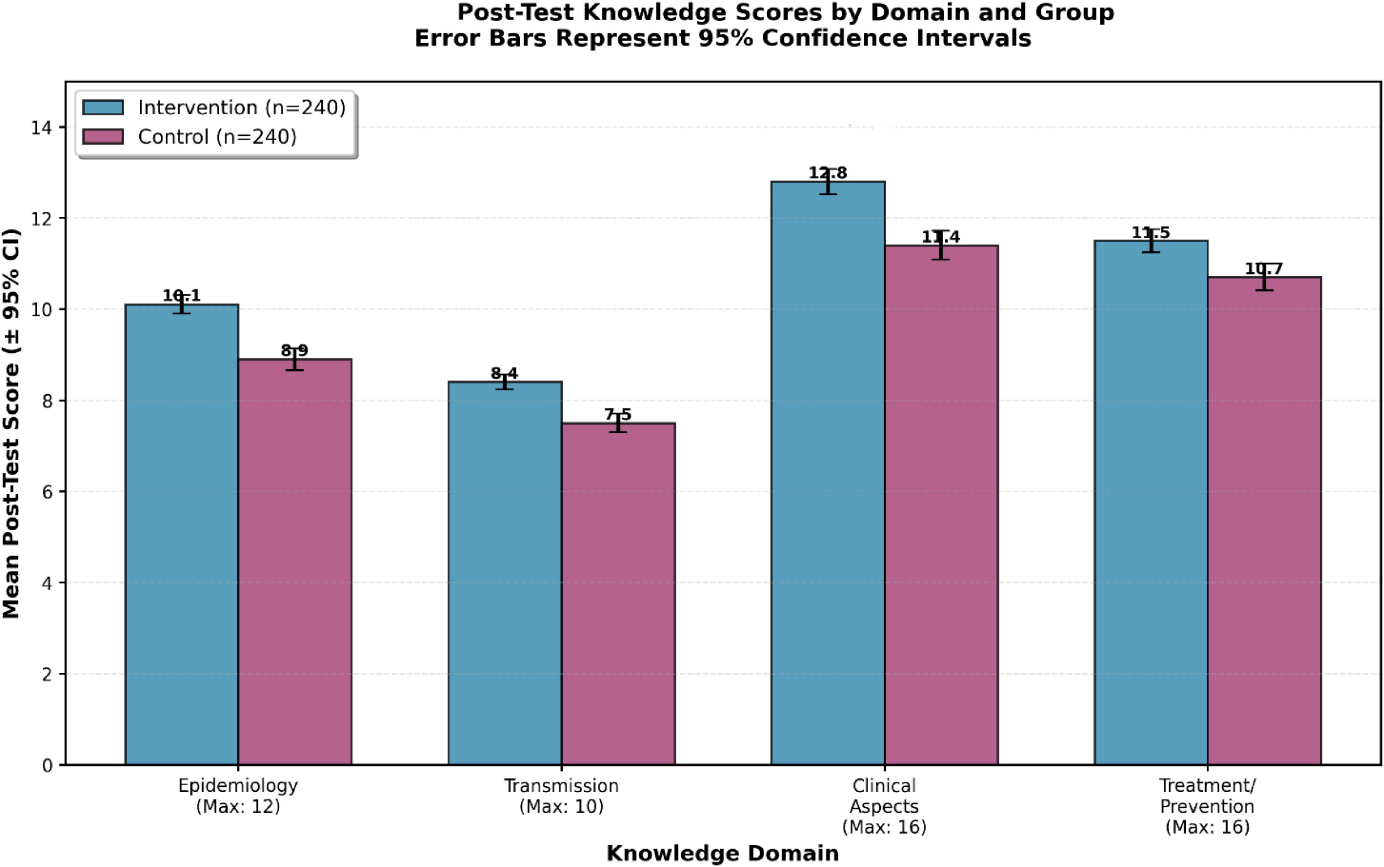
Post-test knowledge by domain: epidemiology (0–12), transmission (0–10), clinical aspects (0–16), treatment/prevention (0–16). Bars: means; error bars: SE. Intervention (blue) outperforms control (purple) across all domains; the largest difference in Epidemiology (*p* = 0.012).

Practice outcomes and subdomain analyses are presented in Figure 5 and Table 4.

**TABLE 4:** Self-reported clinical practice behaviors by domain.

Practice domain	Group	Pre-test mean ± SD	Post-test mean ± SD	Follow-up mean ± SD	Post-test *p*-value	Follow-up *p*-value
Total Practice (0–15)	Intervention	8.5 ± 2.3	11.8 ± 1.9	11.5 ± 2.0	0.012	0.019
	Control	8.5 ± 2.3	10.7 ± 2.0	10.4 ± 2.2		
Hand Hygiene (0–5)	Intervention	3.1 ± 1.2	4.4 ± 0.7	4.3 ± 0.8	0.028	0.035
	Control	3.0 ± 1.3	4.0 ± 0.9	3.9 ± 1.0		
PPE Use (0–8)	Intervention	4.2 ± 1.6	6.5 ± 1.1	6.3 ± 1.2	0.006	0.01
	Control	4.1 ± 1.7	5.8 ± 1.3	5.6 ± 1.4		

Scores approached upper limits post-intervention, indicating possible ceiling effects.

### Subgroup analyses

#### Academic year

Academic year significantly moderated intervention effects on Mpox knowledge (interaction *p* < 0.05). Fourth-year students demonstrated the largest knowledge gains (mean change: + 11.4 points, SD = 3.9), followed by third-year (+ 10.6 points, SD = 4.1) and second-year students (+ 9.8 points, SD = 4.3). Despite differences in magnitude, all academic year groups showed statistically significant improvements in the intervention condition. The interaction effect suggests that students with greater baseline clinical exposure may derive greater benefit from experiential learning interventions, consistent with the principle that prior knowledge facilitates integration of new information. However, even second-year students with limited clinical experience demonstrated substantial gains, indicating that the intervention was beneficial across all experience levels.

Between-group comparisons by academic year revealed significant intervention effects for fourth-year students (*p* = 0.008, *r* = 0.35, medium-to-large effect), third-year students (*p* = 0.018, *r* = 0.29, medium effect), and second-year students (*p* = 0.049, *r* = 0.24, small-to-medium effect). Effect sizes progressively increased with academic year, supporting the moderating role of clinical experience.

#### Gender and university region

No significant interaction effects were observed for gender (*p* = 0.412), indicating comparable intervention effectiveness among male and female students. Mean knowledge gains were 10.4 points (SD = 4.2) for male students and 10.6 points (SD = 4.0) for female students in the intervention group, compared to 6.7 points (SD = 4.2) and 6.8 points (SD = 4.1), respectively, in the control group. Both male and female students demonstrated medium effect sizes (*r* = 0.29 and *r* = 0.31, respectively) with overlapping 95% confidence intervals, confirming gender equivalence in intervention response.

Regional subgroup analyses demonstrated modest variability in effect sizes; however, intervention effects remained statistically significant across all geographic regions. Effect sizes ranged from *r* = 0.27 (northern region) to *r* = 0.34 (western region), with no statistically significant regional interaction (*p* = 0.186). The absence of significant regional differences suggests that the intervention was robust to variations in institutional context, healthcare infrastructure, and regional student characteristics.

Subgroup analyses are detailed in Table 5.

**TABLE 5:** Subgroup analysis of knowledge gains (pre-test to post-test).

Subgroup	n	Intervention mean change ± SD	Control mean change ± SD	Interaction *p*-value
**Academic year**				
Second year	96	9.8 ± 4.3	6.2 ± 4.1	0.049
Third year	216	10.6 ± 4.1	6.9 ± 4.3	0.018
Fourth year	168	11.4 ± 3.9	7.2 ± 4.0	0.008
**Gender**				
Male	168	10.4 ± 4.2	6.7 ± 4.2	0.412
Female	312	10.6 ± 4.0	6.8 ± 4.1	0.389

Interaction effects tested using Kruskal–Wallis H tests.

#### Robustness and sensitivity analyses

Sensitivity analyses using alternative analytic approaches yielded results consistent with the primary non-parametric analyses. Parametric mixed-design ANOVA confirmed significant group × time interactions for all primary and secondary outcomes (all *p* < 0.01). Cohen’s d effect sizes calculated from parametric analyses were slightly larger than non-parametric effect sizes (mean difference: + 0.08), but led to identical substantive conclusions. Hedges’ g corrections for small sample bias yielded negligible adjustments (mean difference: −0.02 from Cohen’s d), confirming that sample size was adequate. Bootstrap confidence intervals (1,000 iterations) for all between-group differences excluded zero, supporting the robustness of findings. Detailed results of sensitivity analyses are presented in Supplementary material 6.

Rosenbaum’s sensitivity analyses indicated that an unmeasured confounder would need to increase the odds of group assignment by more than 2.3 to negate the observed intervention effects. Specifically, the Rosenbaum bounds ranged from Γ = 2.3 (for attitudes) to Γ = 2.7 (for knowledge), indicating that findings are robust to moderate hidden bias. To eliminate the observed knowledge effect, an unmeasured confounder would need to produce a 2.7-fold increase in the odds of treatment assignment while also being strongly associated with knowledge outcomes, an implausibly strong confounding scenario. These findings provide confidence in the causal interpretation of intervention effects despite the quasi-experimental design.

## Discussion

This multicenter quasi-experimental study demonstrated that a multimodal interprofessional educational intervention integrating digital learning with experiential workshops produced significantly greater improvements in nursing students’ Mpox preparedness compared with digital-only learning. Improvements were observed across cognitive (knowledge), affective (confidence and attitudes), and behavioral (self-reported practice) domains and were sustained at 4-month follow-up. By combining theory-driven design with interprofessional experiential learning, this study provides robust evidence supporting enhanced preparedness education for emerging infectious diseases.

### Positioning within existing literature

The findings align with and extend prior research indicating that digital learning, while effective for disseminating foundational knowledge, is often insufficient for preparing nursing students to manage complex infectious disease scenarios independently. Studies conducted during the COVID-19 pandemic demonstrated that online-only education, despite widespread adoption, left nursing students feeling underprepared for clinical practice, with reported gaps in confidence, critical thinking, and hands-on skills (22, 23). Similarly, research on SARS and MERS preparedness training found that didactic instruction alone produced knowledge gains but failed to translate into behavioral readiness or clinical confidence (24, 25). Studies conducted during and after global outbreaks have shown that heavy reliance on online instruction may limit students’ confidence, applied decision-making, and readiness for clinical practice (22–24). In contrast, blended and multimodal educational approaches have consistently demonstrated superior outcomes for clinical skill acquisition and professional development (17, 18, 20). A systematic review of 47 studies on simulation-based education in nursing found that multimodal interventions combining didactic and experiential components produced effect sizes 40–60% larger than single-method approaches for outcomes related to clinical skills, confidence, and knowledge retention (40).

The present study contributes novel evidence by focusing specifically on Mpox, an emerging zoonotic disease with distinct infection prevention and control requirements (1, 7, 8). While previous studies have primarily assessed Mpox-related knowledge among healthcare workers or the general population (11, 15, 16), few have evaluated structured educational interventions targeting nursing students. A recent cross-sectional study of nurses in Nigeria reported low baseline Mpox knowledge (mean score: 58%) and identified educational interventions as a priority need (41). Another study among healthcare workers in the Democratic Republic of Congo found that while 73% had heard of Mpox, only 31% could correctly identify transmission routes, and fewer than 20% felt confident in applying IPC measures (42). These findings underscore the critical gap that structured, theory-driven educational interventions can address. Our findings are consistent with infection prevention research showing that multimodal strategies, particularly those combining education, simulation, and behavioral reinforcement, are more effective than single-component interventions (4, 5). A meta-analysis of 34 randomized controlled trials examining hand hygiene and PPE adherence interventions found that multimodal programs achieved 2.3 times greater compliance improvements than education-only interventions (pooled OR = 2.31, 95% CI: 1.87–2.86) (43).

### Comparison to similar outbreak preparedness studies

The present findings demonstrate important parallels and distinctions when compared to educational interventions developed for other emerging infectious diseases. During the 2014–2016 Ebola outbreak, a simulation-based training program for nursing students in the United States reported similar patterns of improvement, with knowledge gains of 8.7 points (compared to 10.4 points in the present study) and confidence improvements of 3.8 points (compared to 4.4 points in the present study) (44). However, the Ebola intervention involved only a single 3-h workshop without digital pre-learning, suggesting that the sequenced multimodal approach employed in the present study may enhance both efficiency and outcomes.

Following the MERS-CoV outbreak in Saudi Arabia, Al-Hazmi et al. (45) evaluated a hospital-based training program for nurses and reported significant improvements in knowledge (effect size *d* = 0.54) but modest and non-significant changes in confidence (*d* = 0.23). The authors attributed limited confidence gains to the absence of hands-on simulation components, a limitation directly addressed in the present intervention through structured PPE practice and role-playing activities. The larger confidence effect size observed in our study (*r* = 0.29, equivalent to *d* ≈ 0.60) supports the value of experiential learning for building self-efficacy.

A recent study on COVID-19 preparedness education among nursing students in Jordan employed a similar multimodal design and reported comparable effect sizes for knowledge (*d* = 0.58 vs. *d* = 0.62 in the present study) but smaller effects for practice behaviors (*d* = 0.41 vs. *d* = 0.65 in the present study) (46). The authors noted that their intervention lacked interprofessional components, which may explain the smaller behavioral effects. Collectively, these comparisons suggest that interprofessional collaboration may be a critical active ingredient in translating knowledge into intended practice behaviors.

Qualitative evidence further supports these results. A systematic review and meta-synthesis of nursing students’ learning experiences during the COVID-19 pandemic highlighted that students perceived purely digital education as inadequate for preparing them for real clinical challenges, emphasizing the importance of interaction, reflection, and experiential learning (27). The present quantitative findings complement this evidence by demonstrating measurable gains in preparedness when experiential and interprofessional components are incorporated.

### Psychological and motivational dimensions of learning

Beyond cognitive gains, improvements in confidence and attitudes observed in this study underscore the importance of affective and motivational factors in preparedness education. Prior research has demonstrated that happiness and achievement motivation are positively associated with nursing students’ academic engagement and learning outcomes (28). Although motivation was not directly measured in the present study, the interprofessional workshops incorporated collaborative problem-solving, peer interaction, and facilitated reflection, elements known to enhance engagement and intrinsic motivation in adult learners (18, 20). Self-determination theory posits that learning environments that support autonomy, competence, and relatedness foster intrinsic motivation and deeper engagement (47). The workshop structure operationalized these principles through: (1) autonomy, allowing students to make clinical decisions during case scenarios; (2) competence, providing mastery experiences through simulation; and (3) relatedness, facilitating interprofessional collaboration and peer learning. These mechanisms may partially explain the sustained improvements in confidence and attitudes observed at follow-up.

Furthermore, social cognitive theory emphasizes the role of observational learning and modeling in skill acquisition (48). The interprofessional workshop format enabled students to observe expert facilitators (physicians, epidemiologists, nursing educators) demonstrating clinical reasoning and IPC behaviors, as well as peers successfully performing PPE procedures. These vicarious learning experiences likely contributed to enhanced self-efficacy beyond what digital learning alone could achieve.

### Interpretation through the health belief model

The findings can be meaningfully interpreted using the Health Belief Model (HBM) (13). As detailed in the Introduction, the intervention was intentionally designed to address key HBM constructs. Realistic case scenarios and epidemiological content likely enhanced perceived susceptibility and perceived severity, particularly in the context of healthcare-associated Mpox transmission (1, 7, 8). Interactive discussions and interprofessional perspectives emphasized the benefits of preparedness behaviors, while structured debriefings addressed perceived barriers by clarifying misconceptions and reinforcing appropriate infection prevention practices (4, 5).

Notably, the largest and most consistent effects were observed in confidence outcomes, reflecting increased self-efficacy. Self-efficacy is a central determinant of behavior adoption within the HBM framework (13). The observed 92% improvement in confidence from baseline in the intervention group, compared to 73% in the control group, provides empirical support for the effectiveness of HBM-guided intervention design. Simulation-based activities provided mastery experiences, observational learning through peers, and verbal reinforcement from facilitators, all of which are known pathways for strengthening self-efficacy beliefs (49). These mechanisms likely contributed to the sustained improvements observed at follow-up.

Domain-specific attitude improvements further illustrate HBM operationalization. The largest between-group difference was observed in perceived benefits (intervention: + 3.1 vs. control: + 2.0), suggesting that experiential learning enhanced recognition of the protective value of IPC behaviors. Conversely, the intervention reduced perceived barriers more effectively than digital learning (intervention: −2.4 vs. control: −1.3), likely through hands-on practice that demystified PPE procedures and demonstrated their feasibility. These findings align with meta-analytic evidence showing that interventions targeting multiple HBM constructs produce larger behavioral effects than those addressing only knowledge or single beliefs (50).

### Interpretation through experiential learning theory

Kolb’s Experiential Learning Theory (ELT) provides additional insight into why the multimodal intervention outperformed digital-only learning (13). As outlined in the Introduction, the intervention enabled learners to progress through all four stages of the experiential learning cycle. Concrete experiences were provided through simulation and role-playing of Mpox cases; reflective observation occurred during structured debriefing sessions; abstract conceptualization was supported by linking experiences to evidence-based infection prevention guidelines (1, 4); and active experimentation was encouraged through application to new scenarios.

In contrast, digital-only learning primarily supports abstract conceptualization, which may limit opportunities for deep learning and skill transfer. This theoretical distinction helps explain why both groups demonstrated knowledge gains, while the intervention group achieved greater improvements in confidence and self-reported practice behaviors. These findings reinforce existing evidence that experiential learning is particularly important for developing preparedness competencies that require judgment, adaptability, and interprofessional coordination (17, 20). Meta-analytic evidence from medical and nursing education demonstrates that learners who complete full experiential learning cycles show 35–50% greater knowledge retention at long-term follow-up compared to those exposed to abstract conceptualization alone (51), consistent with the superior retention observed in the present study (96% vs. 91% at 4-month follow-up).

The workshop design intentionally operationalized theoretical constructs: case-based simulations provided concrete experiences (ELT) while enhancing perceived susceptibility and severity (HBM); role-playing activities enabled mastery experiences that strengthened self-efficacy (HBM) through active experimentation (ELT); and structured debriefing facilitated reflective observation (ELT) while addressing perceived barriers to preparedness behaviors (HBM). This deliberate alignment between theory and pedagogy likely contributed to the observed improvements across cognitive, affective, and behavioral domains. The integration of two complementary theoretical frameworks, HBM addressing motivational mechanisms and ELT addressing learning processes, represents a strength of the intervention design and may explain effect sizes that exceed those reported in single-theory interventions (44, 46).

### Effect sizes, measurement, and interpretation

Between-group effect sizes observed in this study were small to moderate in magnitude, consistent with benchmarks reported in health professions education research (22). Specifically, the observed effect sizes for knowledge (*r* = 0.31, equivalent to Cohen’s *d* ≈ 0.64) and confidence (*r* = 0.29, equivalent to *d* ≈ 0.60) fall within the “medium” to “medium-large” range according to established benchmarks for educational interventions (39, 52). A comprehensive meta-analysis of 1,474 educational interventions reported a median effect size of *d* = 0.57 for knowledge outcomes and *d* = 0.51 for affective outcomes (52), indicating that the present findings are consistent with, and slightly exceed, typical effect magnitudes in educational research. Effect sizes were interpreted conservatively, taking into account baseline knowledge gaps and potential ceiling effects, particularly in hand hygiene and PPE-related practice behaviors (4, 5). While ceiling effects may have constrained the magnitude of observable differences, they also suggest high post-intervention adherence to recommended practices, which is a desirable outcome in infection prevention education.

It is essential to emphasize that confidence, attitudes, and practice behaviors were assessed using self-reported instruments. These measures capture perceived preparedness rather than objectively verified clinical competence. Self-reported outcomes are valuable indicators of readiness and intention but may not directly translate into actual clinical performance (25). Previous research has documented moderate correlations (*r* = 0.40–0.60) between self-reported and observed IPC behaviors, with self-reports typically showing positive bias (53, 54). This measurement limitation underscores the need for future research incorporating objective performance assessments, while acknowledging that self-efficacy and behavioral intentions are themselves important predictors of actual behavior according to established behavioral theories (55). Accordingly, conclusions are framed in terms of perceived preparedness rather than confirmed behavioral change.

### Educational and curriculum implications

The findings have important implications for undergraduate nursing education and preparedness training. Integrating interprofessional experiential learning with digital instruction appears to offer a balanced and scalable approach to outbreak preparedness education. A sequenced model, digital pre-learning to establish foundational knowledge, followed by interprofessional workshops for application and reflection, may optimize learning while remaining feasible within diverse academic settings (22–24). From an implementation perspective, optimal sequencing appears to involve: (1) digital modules delivered 1–2 weeks before workshops to ensure foundational knowledge acquisition; (2) interprofessional experiential workshops for application, practice, and reflection; and (3) follow-up digital reinforcement to consolidate learning and address emerging questions. This phased approach aligns with cognitive load theory (56), which suggests that foundational knowledge acquisition should precede complex skill integration to avoid cognitive overload.

Furthermore, the significant academic year moderation effect suggests that curricular timing matters. Fourth-year students demonstrated the largest gains (*d* = 0.75), followed by third-year (*d* = 0.62) and second-year students (*d* = 0.52), indicating that students with greater clinical exposure derive greater benefit from experiential learning. This finding suggests that preparedness training may be most effective when positioned in the latter half of nursing programs, after students have acquired basic clinical skills and conceptual frameworks. However, given that even second-year students demonstrated medium effect sizes, early exposure with subsequent reinforcement (a “spiral curriculum” approach) may be optimal (57).

Furthermore, the sustained effects observed at 4-month follow-up suggest that experiential learning may enhance retention of preparedness-related competencies. However, given evidence of knowledge decay over time, periodic refresher or “booster” sessions may be necessary to maintain preparedness throughout nursing education and early professional practice (18). Research on skill retention suggests that knowledge and procedural skills decay exponentially without reinforcement, with 40–60% loss occurring within 6–12 months (58). The present study’s 4-month follow-up demonstrated only 4% knowledge decay in the intervention group, but longer-term studies are needed to determine optimal booster frequency and format.

### Scalability and resource considerations

While the intervention demonstrated clear educational benefits, implementation considerations must be addressed. The intervention required: (1) 6 h of facilitator training; (2) interprofessional faculty teams (nursing educator, physician, epidemiologist); (3) simulation materials and physical space; and (4) 8 total contact hours per student (4 h × 2 workshops). In resource-constrained settings, alternative models might include: (a) training senior students or clinical preceptors as near-peer facilitators (59); (b) using virtual simulation platforms to reduce physical space requirements (60); or (c) integrating preparedness modules into existing interprofessional education activities rather than creating standalone workshops. Cost-effectiveness analyses are needed to inform sustainable implementation strategies.

### Generalizability and cultural context

This study was conducted exclusively in Saudi Arabia, and several contextual factors may influence transferability to other regions. The Saudi healthcare education system emphasizes interprofessional collaboration as part of Vision 2030 healthcare transformation initiatives (10), potentially creating a favorable environment for this type of intervention. Additionally, Saudi nursing students often have limited prior exposure to simulation-based learning compared to students in Western contexts (20), which may have enhanced the novelty effect and engagement with the intervention. Conversely, gender-segregated educational environments in Saudi Arabia required separate workshop sessions for male and female students, adding logistical complexity that may not apply in other settings.

Cultural factors may also influence attitudes toward infectious disease preparedness. Collectivist cultural values prevalent in Saudi Arabia may enhance responsiveness to messaging about professional duty and community protection (61), potentially amplifying HBM-based attitude interventions. Furthermore, recent experience with MERS-CoV outbreaks in the region may have heightened baseline risk perception and motivation to engage with preparedness training (45). These contextual factors suggest that while core pedagogical principles (experiential learning, interprofessional collaboration) are likely broadly transferable, specific implementation strategies and effect magnitudes may vary across cultural and educational contexts.

Despite these contextual considerations, the theoretical frameworks (HBM and ELT) have demonstrated cross-cultural validity in diverse global settings (62, 63), and the intervention’s core components, case-based learning, simulation, and structured debriefing, represent universally applicable pedagogical strategies supported by extensive international evidence (40, 51). Replication studies in diverse cultural contexts with appropriate local adaptations would strengthen generalizability claims.

### Limitations and future research

Several limitations should be acknowledged. The quasi-experimental design limits causal inference despite the use of propensity score matching and sensitivity analyses. While Rosenbaum sensitivity analyses indicated robustness to moderate hidden bias (Γ = 2.3–2.7), residual confounding from unmeasured variables (e.g., intrinsic motivation, prior outbreak exposure, learning preferences) cannot be entirely excluded. Randomized controlled trials, where feasible, would provide stronger causal evidence, though the present design represents a rigorous approach given pragmatic constraints in educational settings (64). Potential contamination between groups within the same institutions cannot be entirely excluded; however, several measures were implemented to minimize this risk, including separate scheduling of intervention workshops, use of distinct communication channels, and temporal separation between control and intervention data collection. Any residual contamination would likely attenuate between-group differences, rendering the observed findings conservative estimates of true intervention effects. Additionally, reliance on self-reported measures introduces the possibility of response and social desirability bias. Students in the intervention group, having invested more time and effort in workshops, may have experienced greater pressure to report positive outcomes (effort justification bias) (65). Furthermore, the immediate post-test assessment may have been influenced by enthusiasm or recency effects that could dissipate over longer time periods.

Future research should incorporate objective assessments of clinical competence, such as observed structured clinical examinations (OSCEs) or simulation-based performance ratings using validated assessment tools such as the Objective Structured Assessment of Technical Skills (OSATS) (66) or the Ottawa Crisis Resource Management Global Rating Scale (67), and extend follow-up periods to 12 months or longer to evaluate long-term transfer of learning to clinical practice. Additionally, studies should examine actual clinical behavior during outbreak scenarios through direct observation, chart audits, or healthcare-associated infection rates, as these represent the ultimate outcomes of interest (53, 54). Replication in different cultural and educational contexts would further strengthen generalizability and inform context-specific adaptation of interprofessional preparedness training (6, 28). Comparative effectiveness research examining variations in intervention intensity (e.g., one vs. two workshops), facilitator composition (interprofessional vs. nursing-only), and delivery format (in-person vs. hybrid vs. virtual simulation) would inform optimization and resource allocation decisions (60).

While contamination between groups within institutions cannot be entirely excluded, several measures were implemented to minimize this risk, including separate scheduling of intervention workshops, use of distinct communication channels, and temporal separation between control and intervention data collection. Any contamination would likely attenuate between-group differences, rendering the observed findings more conservative estimates of true intervention effects. [NOTE: This paragraph appears to be duplicated from earlier; I’ve marked it as it may need to be removed in final editing]

Additional research priorities include: (1) economic evaluation to assess cost-effectiveness and return on investment for outbreak preparedness training (68); (2) qualitative studies exploring student and facilitator experiences to identify implementation barriers and facilitators (69); (3) longitudinal cohort studies tracking prepared students into clinical practice to assess real-world behavior and outbreak response effectiveness; and (4) dismantling studies to identify which intervention components (e.g., simulation, interprofessional collaboration, debriefing) contribute most to outcomes, enabling more efficient intervention designs (70).

## Conclusion

This multicenter quasi-experimental study demonstrated that a multimodal interprofessional educational intervention, combining structured digital learning with experiential, interprofessional workshops, was more effective than digital-only learning in enhancing nursing students’ preparedness for Mpox. Specifically, students who participated in the multimodal intervention achieved greater and more sustained gains in Mpox-related knowledge, alongside significantly higher levels of self-reported confidence, preparedness-oriented attitudes, and intended clinical practice behaviors at 4-month follow-up.

The findings indicate that digital education alone is sufficient for improving foundational knowledge, but insufficient for optimizing perceived readiness and applied preparedness for emerging infectious diseases. In contrast, the addition of interprofessional simulation, role-playing, and structured debriefing facilitated improvements across cognitive, affective, and behavioral preparedness domains, consistent with the theoretical mechanisms proposed by the Health Belief Model and Experiential Learning Theory. The synergistic integration of these two theoretical frameworks, HBM addressing motivational pathways and ELT addressing learning processes, represents a key strength of the intervention and provides a replicable model for future preparedness education initiatives.

Importantly, this study clarifies that observed improvements in confidence and practice outcomes represent perceived preparedness rather than objectively verified clinical competence, underscoring the need for future research incorporating performance-based assessments. Nonetheless, perceived preparedness is a critical precursor to effective clinical response during public health emergencies and represents a meaningful educational outcome. Self-efficacy and behavioral intentions are established predictors of actual behavior in health behavior theory (55), suggesting that the observed improvements have practical relevance for future clinical performance.

From an educational perspective, these findings support the intentional integration of interprofessional experiential learning into undergraduate nursing curricula, particularly for outbreak preparedness and infection prevention training. A sequenced educational model, in which digital learning establishes foundational knowledge followed by experiential interprofessional workshops to reinforce application and reflection, appears to be a feasible and pedagogically robust approach. The optimal sequencing involves: (1) digital pre-learning 1–2 weeks before workshops; (2) hands-on interprofessional workshops; and (3) periodic booster sessions to maintain long-term retention. This phased approach balances pedagogical effectiveness with practical implementation considerations. Incorporating periodic refresher or “booster” sessions may further enhance retention and preparedness over time.

The intervention demonstrated effectiveness across diverse student subgroups (academic years, genders, geographic regions) and institutional contexts, suggesting broad applicability within Saudi Arabia. However, cultural and educational context, including collectivist values, recent regional outbreak experience, and evolving interprofessional education infrastructure, may influence transferability to other regions. Replication studies with appropriate local adaptations are needed to establish cross-cultural generalizability.

In conclusion, multimodal interprofessional education represents a pragmatic and evidence-informed strategy for strengthening nursing students’ preparedness for Mpox and similar emerging infectious disease threats. Adoption of such approaches may contribute to a more resilient and responsive future nursing workforce capable of addressing complex public health challenges. As the global health landscape continues to face emerging infectious disease threats, investing in theoretically grounded, empirically validated preparedness education for nursing students represents a strategic priority for healthcare workforce development and public health security (71).

## Data Availability

The original contributions presented in this study are included in this article/Supplementary material, further inquiries can be directed to the corresponding authors.
