# Supplementary material for: Multimodal interprofessional education vs. digital learning: enhancing Mpox preparedness in Saudi nursing students
Source: Front Med (Lausanne). 2026 Feb 19;13:1747624. doi: 10.3389/fmed.2026.1747624 (PMC12960114; doi:10.3389/fmed.2026.1747624)
Supplement: Supplementary file 1 [file Table_1.docx]

**SUPPLEMENTARY MATERIALS**

**Supplementary Material S1. Detailed Description of the Multimodal Interprofessional Educational Intervention**

**Overview**

The multimodal interprofessional educational intervention was designed to enhance nursing students’ preparedness for Mpox through the integration of digital learning, experiential simulation, and interprofessional collaboration. The intervention was theoretically grounded in the **Health Belief Model (HBM)** and **Kolb’s Experiential Learning Theory (ELT)** and implemented consistently across all participating institutions.

**Digital Learning Component**

All participants (intervention and control groups) completed four self-directed digital learning modules delivered via the Blackboard Learning Management System. Each module was approximately 60 minutes in duration and included narrated presentations, short instructional videos, and embedded formative quizzes.

**Module 1: Mpox Epidemiology and Transmission**

- Global and regional epidemiology of Mpox
- Modes of transmission (zoonotic, human-to-human, healthcare-associated)
- Risk factors for healthcare workers

**Module 2: Clinical Presentation and Diagnosis**

- Incubation period and symptom progression
- Differential diagnosis (Mpox vs. varicella vs. smallpox)
- Case identification and triage criteria

**Module 3: Infection Prevention and Control (IPC)**

- Standard, contact, and droplet precautions
- Personal protective equipment (PPE) selection
- Environmental cleaning and waste management

**Module 4: Patient Education and Risk Communication**

- Counseling patients and families
- Addressing stigma and misinformation
- Public health reporting responsibilities

Completion was verified through LMS analytics (time-on-task and quiz completion).

**Interprofessional Workshop Component (Intervention Group Only)**

Participants in the intervention group attended **two face-to-face interprofessional workshops**, each lasting four hours, conducted over a two-week period.

**Facilitator Composition and Training**

Each workshop was facilitated by:

- One nursing educator (IPC expertise)
- One infectious disease physician
- One public health epidemiologist

Facilitators underwent a **standardized 6-hour training session** covering:

- Learning objectives and case flow
- Simulation facilitation techniques
- Structured debriefing using ELT principles
- Strategies to ensure consistent delivery across sites

**Workshop Structure**

Each workshop followed the same standardized structure:

1. **Orientation (30 minutes)**
   - Learning objectives
   - Overview of Mpox response protocols
   - Psychological safety and teamwork expectations
2. **Collaborative Case Analysis (90 minutes)**
   - Small interprofessional groups (6–8 students)
   - Unfolding Mpox case scenarios requiring:
     - Risk assessment
     - Isolation decisions
     - Interprofessional coordination
3. **Simulation and Role-Playing (90 minutes)**
   - PPE donning and doffing
   - Patient isolation procedures
   - Risk communication with simulated patients and family members
4. **Structured Debriefing (60 minutes)**
   - Guided reflection aligned with Kolb’s ELT
   - Discussion of emotions, decision-making, and teamwork
   - Translation of experience into clinical principles

**Mapping to Theoretical Frameworks**

| **Intervention Component** | **HBM Construct** | **ELT Stage** |
| --- | --- | --- |
| Case-based risk scenarios | Perceived susceptibility & severity | Concrete experience |
| IPC simulations | Self-efficacy | Active experimentation |
| Group discussion | Perceived benefits | Reflective observation |
| Facilitated debriefing | Barrier reduction | Abstract conceptualization |

**Supplementary Material S2. Detailed Case Scenarios**

**Case Scenario 1: Suspected Mpox in Emergency Department**

- 29-year-old male with fever and vesicular rash
- Recent travel history and close contact exposure
- Students required to:
  - Apply triage protocols
  - Initiate isolation
  - Select appropriate PPE
  - Notify infection control team

**Case Scenario 2: Inpatient Mpox Case**

- Confirmed Mpox diagnosis
- Challenges related to:
  - PPE compliance
  - Family communication
  - Environmental decontamination
  - Interprofessional coordination

**Supplementary Material S3. Structured Debriefing Framework**

Debriefing followed a **four-stage ELT-based model**:

1. **Description** – What happened during the scenario?
2. **Analysis** – Why were specific decisions made?
3. **Conceptualization** – What principles can be generalized?
4. **Application** – How will this be applied in future clinical practice?

Facilitators used standardized prompts to ensure consistency across institutions.

**Supplementary Material S4. Psychometric Properties of Data Collection Instruments**

| **Instrument** | **Items** | **Scale** | **Cronbach’s α** |
| --- | --- | --- | --- |
| Mpox Knowledge Questionnaire | 27 | MCQ | 0.88 |
| Confidence in Mpox Management | 3 | 4-point Likert | 0.89 |
| Attitude Toward Mpox Preparedness | 12 | 7-point Likert | 0.85 |
| Self-Reported Practice Behaviors | 15 | Dichotomous | 0.91 |

**Supplementary Material S5. Sensitivity and Robustness Analyses**

To validate findings, the following analyses were conducted:

- Non-parametric tests (Mann–Whitney U, Wilcoxon signed-rank)
- Bootstrap resampling (1,000 iterations)
- Rosenbaum bounds sensitivity analysis
- Alternative effect size metrics (Hedges’ g, Glass’s Δ)

All analyses yielded conclusions consistent with primary results.

**Table S1. Theoretical Mapping of Multimodal Interprofessional Educational Intervention Components to Health Belief Model (HBM) Constructs and Experiential Learning Theory (ELT) Stages with Corresponding Learning Objectives**

| **Intervention Component** | **Specific Activities** | **Duration** | **HBM Construct(s) Targeted** | **ELT Stage(s)** | **Specific Learning Objectives** |
| --- | --- | --- | --- | --- | --- |
| **DIGITAL LEARNING MODULES** (Both Groups) |  |  |  |  |  |
| **Module 1: Mpox Epidemiology and Transmission** | • Narrated presentations on global/regional epidemiology • Video demonstrations of transmission routes • Interactive maps showing outbreak patterns • Formative quizzes | 60 min | • Perceived Susceptibility • Perceived Severity | • Abstract Conceptualization | 1. Describe the global and regional epidemiology of Mpox 2. Identify all modes of transmission (zoonotic, human-to-human, healthcare-associated) 3. Recognize risk factors for healthcare workers 4. Analyze outbreak patterns and high-risk populations |
| **Module 2: Clinical Presentation and Diagnosis** | • Narrated presentations on clinical features • Photo galleries of rash progression • Differential diagnosis algorithms • Case vignettes with diagnostic reasoning • Formative quizzes | 60 min | • Perceived Severity • Perceived Susceptibility | • Abstract Conceptualization | 1. Identify incubation period and symptom progression 2. Differentiate Mpox from varicella and smallpox 3. Apply case identification and triage criteria 4. Recognize atypical presentations and complications |
| **Module 3: Infection Prevention and Control (IPC)** | • Narrated presentations on IPC protocols • Video demonstrations of PPE procedures • Guidelines for environmental cleaning • Waste management protocols • Formative quizzes | 60 min | • Perceived Benefits • Perceived Barriers • Self-efficacy (initial cognitive level) | • Abstract Conceptualization | 1. Describe standard, contact, and droplet precautions for Mpox 2. Select appropriate PPE for different clinical scenarios 3. Explain environmental cleaning and waste management procedures 4. Identify barriers to IPC compliance and mitigation strategies |
| **Module 4: Patient Education and Risk Communication** | • Communication frameworks • Videos on counseling techniques • Stigma reduction strategies • Public health reporting procedures • Formative quizzes | 60 min | • Perceived Benefits • Perceived Barriers (addressing stigma) | • Abstract Conceptualization | 1. Demonstrate effective patient and family counseling techniques 2. Address stigma and misinformation using evidence-based approaches 3. Explain public health reporting responsibilities 4. Apply risk communication principles in diverse scenarios |
| **INTERPROFESSIONAL WORKSHOP COMPONENTS** (Intervention Group Only) |  |  |  |  |  |
| **Workshop Session 1** |  |  |  |  |  |
| **Orientation and Team Formation** | • Introduction to learning objectives • Overview of Mpox response protocols • Establishment of psychological safety • Formation of interprofessional teams (6-8 students) • Discussion of teamwork expectations | 30 min | • Professional Responsibility • Perceived Benefits (of interprofessional collaboration) | • Concrete Experience (initial) • Abstract Conceptualization | 1. Articulate the role of interprofessional collaboration in outbreak response 2. Establish norms for effective teamwork and communication 3. Recognize the complementary expertise of nursing, medicine, and public health 4. Commit to active participation and peer learning |
| **Collaborative Case Analysis: Emergency Department Scenario** | • Small group analysis of unfolding case: - 29-year-old male with fever and vesicular rash - Recent travel history - Close contact exposure • Interprofessional discussion of: - Risk assessment - Triage decisions - Isolation protocols - Notification procedures • Case progression with new clinical data • Group decision-making under time pressure | 45 min | • Perceived Susceptibility (healthcare-associated transmission) • Perceived Severity (clinical complications) • Perceived Benefits (early identification) • Self-efficacy (decision-making) | • Concrete Experience • Reflective Observation • Active Experimentation | 1. Apply triage protocols to suspected Mpox cases 2. Make evidence-based isolation decisions collaboratively 3. Coordinate interprofessional team responses 4. Prioritize competing clinical demands in emergency settings 5. Communicate risk effectively to team members |
| **Collaborative Case Analysis: Inpatient Management Scenario** | • Small group analysis of confirmed case: - Inpatient with confirmed Mpox diagnosis - Challenges in PPE compliance - Family communication needs - Environmental decontamination issues • Discussion of: - Sustained IPC adherence - Patient and family education - Interprofessional coordination - Ethical considerations | 45 min | • Perceived Barriers (compliance challenges) • Perceived Benefits (sustained IPC) • Self-efficacy (problem-solving) • Professional Responsibility | • Concrete Experience • Reflective Observation • Active Experimentation | 1. Implement sustained IPC measures for hospitalized patients 2. Address PPE compliance barriers in clinical teams 3. Provide culturally sensitive patient and family education 4. Coordinate environmental decontamination procedures 5. Navigate ethical dilemmas in isolation care |
| **Simulation: PPE Donning and Doffing** | • Hands-on practice with actual PPE: - Gown, gloves, N95 respirator, face shield, shoe covers • Supervised practice of correct sequence • Peer observation and feedback • Error identification and correction • Timed practice for efficiency • Facilitator assessment using checklist | 40 min | • Self-efficacy (mastery experience) • Perceived Barriers (technical difficulty) • Perceived Benefits (protective effectiveness) | • Concrete Experience • Active Experimentation | 1. Demonstrate correct PPE donning sequence for Mpox care 2. Perform safe PPE doffing to prevent self-contamination 3. Identify and correct common PPE errors 4. Achieve proficiency within recommended time standards 5. Apply infection prevention principles during PPE use |
| **Simulation: Patient Isolation Procedures** | • Role-playing scenario: - Student as nurse - Simulated patient with Mpox - Facilitator as family member • Practice of: - Room entry/exit procedures - Specimen collection - Medication administration - Vital sign assessment - Waste disposal • Real-time facilitator observation | 30 min | • Self-efficacy (mastery experience) • Perceived Barriers (workflow integration) • Professional Responsibility | • Concrete Experience • Active Experimentation | 1. Execute safe room entry and exit procedures 2. Perform clinical care tasks while maintaining IPC precautions 3. Integrate infection prevention into routine nursing care 4. Manage clinical workflow efficiently under isolation conditions 5. Maintain patient dignity and therapeutic communication |
| **Simulation: Risk Communication with Simulated Patients** | • Role-playing scenarios: - Explaining diagnosis to anxious patient - Addressing family concerns - Correcting misinformation - Discussing isolation requirements • Use of standardized patients or facilitators • Focus on communication skills and empathy | 20 min | • Perceived Benefits (patient adherence) • Perceived Barriers (stigma) • Self-efficacy (communication) | • Concrete Experience • Active Experimentation | 1. Deliver clear, compassionate explanations of Mpox diagnosis 2. Address patient and family emotional responses effectively 3. Counter stigma and misinformation with evidence 4. Negotiate isolation procedures while preserving autonomy 5. Apply health literacy principles in patient education |
| **Structured Debriefing: Workshop 1** | • Four-stage ELT-based debriefing: **Stage 1 - Description:** What happened? **Stage 2 - Analysis:** Why did you make those decisions? **Stage 3 - Conceptualization:** What principles can be generalized? **Stage 4 - Application:** How will you apply this in practice? • Facilitator-guided reflection • Peer feedback and discussion • Emotional processing • Link to theoretical frameworks | 45 min | • Perceived Barriers (identification and problem-solving) • Perceived Benefits (reflective learning) • Self-efficacy (vicarious learning from peers) | • Reflective Observation • Abstract Conceptualization | 1. Reflect critically on clinical decisions and teamwork 2. Articulate rationale for infection prevention actions 3. Generalize lessons learned to future clinical situations 4. Identify personal learning needs and growth areas 5. Integrate emotional responses into professional development |
| **Workshop Session 2** (Two weeks after Workshop 1) |  |  |  |  |  |
| **Review and Reconnection** | • Brief review of key concepts from Workshop 1 • Sharing of interim reflections • Questions and clarifications • Reactivation of learning mindset | 15 min | • Perceived Benefits (reinforcement) • Self-efficacy (confidence building) | • Reflective Observation • Abstract Conceptualization | 1. Synthesize key learnings from previous workshop 2. Articulate evolving understanding of Mpox preparedness 3. Connect prior learning to new scenarios 4. Demonstrate knowledge retention and integration |
| **Advanced Collaborative Case Analysis: Outbreak Scenario** | • Complex multi-patient scenario: - Multiple suspected cases in ED - Limited isolation rooms - Staffing constraints - Media pressure - Public health coordination • System-level decision-making • Resource allocation dilemmas • Crisis communication | 60 min | • Perceived Susceptibility (outbreak risk) • Perceived Severity (system strain) • Perceived Benefits (coordinated response) • Self-efficacy (crisis management) • Professional Responsibility | • Concrete Experience • Reflective Observation • Active Experimentation | 1. Prioritize resource allocation during outbreak scenarios 2. Coordinate responses across multiple healthcare disciplines 3. Make ethical decisions under resource constraints 4. Communicate effectively with public health authorities 5. Manage personal stress and team morale during crises |
| **Advanced Simulation: Integrated Clinical Scenario** | • Full clinical simulation integrating: - Assessment of suspected case - PPE application - Specimen collection - Isolation implementation - Patient/family communication - Interprofessional coordination • High-fidelity scenario with time pressure • Multiple decision points • Team-based performance | 50 min | • Self-efficacy (complex skill integration) • Perceived Benefits (comprehensive preparedness) • Perceived Barriers (complexity management) | • Concrete Experience • Active Experimentation | 1. Integrate multiple competencies in realistic clinical scenarios 2. Perform under time pressure and uncertainty 3. Coordinate seamlessly with interprofessional team members 4. Adapt to unexpected clinical developments 5. Maintain IPC standards while delivering quality patient care |
| **Structured Debriefing: Workshop 2** | • Enhanced four-stage debriefing: **Stage 1 - Description:** What happened in the complex scenario? **Stage 2 - Analysis:** What factors influenced your performance? **Stage 3 - Conceptualization:** How do these experiences relate to outbreak preparedness principles? **Stage 4 - Application:** How will you maintain preparedness in your future practice? • Emphasis on long-term application • Discussion of ongoing learning strategies • Commitment to future preparedness | 45 min | • Perceived Benefits (long-term preparedness) • Self-efficacy (sustained confidence) • Professional Responsibility (ongoing commitment) | • Reflective Observation • Abstract Conceptualization | 1. Synthesize learning across both workshop sessions 2. Articulate a personal preparedness action plan 3. Identify strategies for maintaining competence over time 4. Commit to ongoing professional development in outbreak preparedness 5. Reflect on professional identity as a prepared healthcare provider |
| **Closure and Future Directions** | • Summary of key learnings • Distribution of reference materials • Discussion of continuing education resources • Commitment to preparedness practice • Post-intervention assessment scheduling | 15 min | • Perceived Benefits (ongoing learning) • Professional Responsibility • Self-efficacy (sustainable confidence) | • Abstract Conceptualization • Active Experimentation (future-oriented) | 1. Consolidate key preparedness competencies 2. Identify reliable resources for ongoing learning 3. Commit to applying learned skills in clinical practice 4. Plan for continued professional development 5. Recognize preparedness as an ongoing professional responsibility |

**Table S1 Legend and Notes:**

**Health Belief Model (HBM) Constructs:**

- **Perceived Susceptibility:** Beliefs about personal vulnerability to Mpox infection, particularly in healthcare settings
- **Perceived Severity:** Beliefs about the seriousness of Mpox and its potential consequences
- **Perceived Benefits:** Beliefs about the effectiveness of preparedness behaviors in reducing risk
- **Perceived Barriers:** Beliefs about obstacles to performing preparedness behaviors (e.g., time, complexity, resources)
- **Self-efficacy:** Confidence in one's ability to successfully perform preparedness behaviors
- **Professional Responsibility:** Beliefs about duty and obligation as a healthcare professional (additional motivational construct)

**Experiential Learning Theory (ELT) Stages:**

- **Concrete Experience:** Direct engagement with learning activities through hands-on practice, simulation, and real-world scenarios
- **Reflective Observation:** Thoughtful consideration of experiences through debriefing, discussion, and guided reflection
- **Abstract Conceptualization:** Development of theories and principles by connecting experiences to evidence-based frameworks
- **Active Experimentation:** Application of learned concepts to new situations and testing of strategies in varied contexts

**Intervention Design Principles:**

1. **Sequential Integration:** Digital modules provided foundational abstract conceptualization before workshops introduced concrete experiences, following principles of cognitive load management
2. **Theory-Practice Alignment:** Each intervention component was explicitly designed to target specific HBM constructs while advancing learners through the ELT cycle
3. **Interprofessional Learning:** Activities intentionally integrated nursing, medical, and public health perspectives to foster collaborative competencies essential for outbreak response
4. **Progressive Complexity:** Workshop 1 focused on individual competencies (PPE, communication) while Workshop 2 emphasized systems-level thinking and integration under crisis conditions
5. **Repetitive Practice:** Core skills (PPE, risk assessment) were practiced multiple times across different contexts to enhance retention and transfer

**Total Intervention Time:**

- Digital Learning: 240 minutes (4 hours) across 4 modules
- Workshop 1: 240 minutes (4 hours)
- Workshop 2: 240 minutes (4 hours)
- **Total: 720 minutes (12 hours)**

**Abbreviations:** ELT, Experiential Learning Theory; HBM, Health Belief Model; IPC, Infection Prevention and Control; Mpox, Monkeypox; PPE, Personal Protective Equipment
